# Reengineering NHS Hospitals in Greece: Redistribution Leads to Rational Mergers

**DOI:** 10.5539/gjhs.v7n5p272

**Published:** 2015-03-16

**Authors:** Athanasios Nikolentzos, Nick Kontodimopoulos, Nikolaos Polyzos, Eleftherios Thireos, Yannis Tountas

**Affiliations:** 1 School of Social Science, Hellenic Open University & Institute for Social and Preventative Medicine, Athens, Greece; 2 School of Social Science, Hellenic Open University & Ministry of Health, Athens, Greece; 3 Department of Social Management, Democritus University of Thrace, Komotini, Greece; 4 Greek National Health System, Athens, Greece; 5 School of Medicine, National and Kapodistrian University of Athens, Athens, Greece

**Keywords:** hospitals, reengineering, efficiency, Greece, mergers

## Abstract

The purpose of this study was to record and evaluate existing public hospital infrastructure of the National Health System (NHS), in terms of clinics and laboratories, as well as the healthcare workforce in each of these units and in every health region in Greece, in an attempt to optimize the allocation of these resources. An extensive analysis of raw data according to supply and performance indicators was performed to serve as a solid and objective scientific baseline for the proposed reengineering of the Greek public hospitals. Suggestions for “reshuffling” clinics and diagnostic laboratories, and their personnel, were made by using a best versus worst outcome indicator approach at a regional and national level. This study is expected to contribute to the academic debate about the gap between theory and evidence based decision-making in health policy.

## 1. Introduction

In the health sector, the task of reorganizing two or more hospitals in an effort to reduce the overall costs of their services has been described as “restructuring”, “reconfiguring”, “reengineering” or more commonly “merging” (Krishnan, 2001; Kjekshus & Hagen, 2007; Ahgren, 2008). Merging does not necessarily result in the closure of one hospital and the extension of another, nor does it imply that a new investment replaces two or more older hospitals. A “merger” may mean that hospital services are reduced in one hospital and concentrated in another so that the former hospital retains only a limited number of specialties or services. Whilst efficiency is obviously the predominant criterion in the merging process, the sensitive nature of health and healthcare requires that policy-makers equally value the usually contradicting (to efficiency) criterion of access equity.

Many motives for mergers have been reported in the literature. One aim is to achieve economic gains, firstly by taking advantage of economies of scale and scope, especially with regard to management costs (Ferguson & Goddard, 1997) and secondly, as a result of rationalizing the provision of services by reducing excess capacity to treat patients. It has also been assumed that clinical quality improves as usage of specialized units increases (Smith, 2001), that quality of medical training increases (Dowie & Gravelle, 1997) and staff recruitment and staff retention become more effective (Ferguson et al., 1997). On the other hand, it has been argued that the evidence for benefits of horizontal mergers is contradictory and often based on subjective beliefs and opinions. Unintended consequences and potential drawbacks of mergers receive less attention. These include disruption of services as a direct consequence of mergers, diseconomies of scale, and problems with staffing, service integration, systems integration, and working practices, as well as issues of equity and access to services (McClenahan, 1999).

The international literature on mergers suggests mixed (or contradictory) results. A series of recent merger retrospective studies which examined the effect of a particular merger have been inconclusive as to the impact on inpatient cost (Haas-Wilson & Garmon, 2011; Tenn, 2011; Thompson, 2011). Other studies having examined the impacts of large numbers of (mostly) private hospital mergers in the US found, in general, little benefit from merger and consolidation (Ho & Hamilton, 2000; Krishnan, 2001; Spang et al., 2001; Town et al., 2006; Dafny, 2009). Case studies of hospital mergers in the UK have highlighted the fact that the pre-merger forecasts of savings in management costs were eventually over-optimistic (Fulop et al., 2002; Hutchings et al., 2003). Finally, a recent UK study examined the impact of mergers on a large set of outcomes including financial performance, productivity, waiting times and clinical quality and found little evidence that mergers achieved gains other than a reduction in activity (Gaynor et al., 2012).

The Greek literature lacks studies on merger efforts in the health sector although many parametric or non-parametric efficiency measurement studies have demonstrated the potential for efficiency gains in Greek hospitals (Aletras, 1999; Athanassopoulos et al., 1999; Athanassopoulos & Gounaris, 2001; Giokas, 2001; Kontodimopoulos et al., 2006; Aletras et al., 2007; Flokou et al., 2011; Kounetas & Papathanassopoulos, 2013; Polyzos, 2013). Empirical evidence is an important factor to guide decision-making, however the existing political and economical situation in Greece, in conjunction to an intense social turmoil, implies that restructuring should rely less on technical and analytical tools, and more on various social, political, geographical and other peculiarities existing nationally and locally in Greece.

## 2. The Situation in Greece

### 2.1 The Economic Crisis

The long-term inefficiencies and shortcomings of the Greek NHS are well known (Tountas et al., 2002; Davaki & Mossialos, 2005; Mossialos & Allin, 2005; Mossialos et al., 2005; Nikolentzos & Mays, 2008; Tountas et al., 2011). Legislative initiatives to confront these inefficiencies have been mostly unsuccessful due to political particularism, fiscal constraints and administrative weaknesses (Tragakes & Polyzos, 1998). Since 2009 the Greek economy has been facing its most severe crisis in recent history and to avert a potential default, the European Commission, the International Monetary Fund and the European Central Bank (referred to as the “Troika”) agreed to a rescue package with immediate bailout loans, and more funds to follow. To secure continuity of the funding, Greece has been required to adopt harsh austerity measures to control its deficit, and their implementation has been constantly monitored and evaluated by the Troika. The Government has been addressing inefficiencies by reducing the size and costs of the public sector, resulting in tax increases and cuts in many areas, including health care. A series of measures have been adopted for the NHS Hospitals such as national tendering procedures for procurement of hospital medical products and pharmaceuticals (Kastanioti et al., 2013), implementation of a DRG-based payment system (Polyzos et al., 2013) and reforms in the pharmaceutical market (Kontodimopoulos et al., 2013; Vandoros & Stargardt, 2013). Hospital mergers captured the public eye and became the centerpiece of health policy reform.

### 2.2 Restructuring Greek Public Hospitals

The formal announcement by the MoH to embark on an effort to restructure NHS hospitals was made in early 2011 when a competent committee was set up (Liaropoulos et al., 2012) to formulate proposals from a managerial prospective in order to decrease administrative costs data. These and relative proposals made by the National School of Public Health were discussed in all Regional Healthcare Authorities (RHA) with their managers and other professional bodies, and the effort concluded in a final text (suggestions from RHA Managers which led to a proposal by the Secretary-General of the Ministry that was examined by the Deputy Ministers, and decisions by the Minister of Health) on 1 July 2011. The Central Council of Regional Healthcare Authorities (including all the above) approved these proposals by the end of October 2011 and after publication of the necessary institutional decisions, these proposals should be implemented by the end of 2011.

The reorganization of the NHS Health Units was undertaken to achieve key objectives relating to:

- development of a new sustainable architecture for optimal allocation of inputs,
- optimal utilization of the public health care system resources,
- efficient and effective operation of the NHS Health Units thus seeking both the unified planning of health services for effective and equitable coverage of citizens by integrated and quality health services, and the necessary economies of scale in rationalization of the health system.

The completion of the hospital’s network reform has led to 82 hospital units out of 131. The remaining 49 have been connected with 80 hospitals as NHS Trusts, whereas 2 not-for-profit public agencies remained autonomous. Additionally five IKA hospitals (white and blue collars’ social insurance fund) were transferred to five main hospitals. The total number of beds of the NHS’s hospitals decreased to 35.360 (+1.990 Psychosocial Rehabilitation Units), from 46.783, while the number of the medical Departments and Units has declined by 600 and the working positions were cut by 15.000 (-20% and -15% respectively). Additionally, changes have been made in the use of eight small hospitals which are turned into urban health centers, support and palliative care units and hospitals for short-term hospitalization and rehabilitation.

## 3. Present Study

### 3.1 Scope

The previously mentioned proposals and decisions were focused mainly on restructuring the management of NHS hospitals, merging some of them and changing the intended use of others. Apart from these important and necessary changes, there is a need for reallocation of medical and laboratory units in order to solve existing problems regarding shortages or oversupply of services in different parts of the country, which generate important inequalities in the distribution of services among the seven Health Regions. For this reason, the scope of the present study was to measure and evaluate the distribution of NHS clinical and laboratory services and workforce in relation to the population in each health region, and to formulate proposals for their proper reallocation.

## 4. Methods

### 4.1 Data Collection

Raw data were collected within a period of six months in 2012 from 129 Greek NHS hospitals (2 hospitals never completed and returned the forms and questionnaires), by the researchers via specially constructed data collection forms and questionnaires, and were subsequently examined and complemented with data from ESY.net, a web-based facility developed by the MoH to collect updated and reliable data from NHS hospitals on a monthly basis. Preliminary analyses were limited to a detailed description of the current situation of the Greek hospital sector. However, it should be taken into account that the study described in this article was carried out on a large scale, and constitutes the second, and most complete, effort to record imaging and diagnostic laboratories, and most importantly available human resources per hospital and per Health Region. This paper exploits the aforementioned data by comparing supply and performance indicators in each Region with the average national scores. Deviations or convergences are used to formulate specific proposals for the reengineering of the public hospital sector or the “reshuffling” of the health professionals and infrastructure at a HR level. The following section provides a detailed description of the supply and performance indicators used in this study.

A. Supply Indicators
A. 1 Hospital beds/1000 population
A. 2 Doctors/1000 population
A. 3 Nurses/1000 population
A. 4 Total staff/1000 population
A. 5 Major Hospital Clinics/100.000 population
A. 6 Major Clinics Hospital beds/1000 population
A. 7 Diagnostic Laboratory Doctors/1000 population
A. 8 Diagnostic Laboratory Nurses/1000 population
A. 9 Diagnostic Laboratory Health workers/1000 population
A. 10 Total Diagnostic Laboratory staff/1000 population
A. 11 Major Hospital Diagnostic Laboratories/100.000 population
B. Performance Indicators
B. 1 Hospital Doctors/Hospital Bed
B. 2 Nurses/Hospital Bed
B. 3 Total staff/Hospital Bed
B. 4 Major clinics’ staff (doctors, nurses and in total)/Hospital Bed

The data were organized according to the following thematic analytical categories:

A. Hospital Clinics (National Health System or University)
1) The type and kind of clinic (National Health System (Note 1) or University)
2) The sector (internal medicine, surgery, psychiatry and intersectorial)
3) Number of hospital beds/ESY clinic-university clinic
4) Number of specialized doctors/ESY clinic-university clinic
5) Number of university doctors/university clinic
6) Number of resident doctors/ESY clinic-university clinic
7) Number of associate – assistant doctors/ESY clinic- university clinic
8) Number of nurse/ESY clinic- university clinic
9) Total number of doctors and nurses/ESY clinic – university clinic
B. Diagnostic Laboratories (ΕSΥ or University)
1) The type and kind of laboratory (National Health System or University)
2) The sector (internal medicine, surgery, psychiatry and intersectorial)
3) Number of specialized doctors/ESY laboratories-university laboratories
4) Number of university doctors/ESY laboratories-university laboratories
5) Number of resident doctors/ESY laboratories-university laboratories
6) Number of associate – assistant doctors/ESY laboratories-university laboratories
7) Number of nurses/ESY laboratories-university laboratories
8) Number of other laboratory health workers/ESY laboratories-university laboratories
9) Total number of doctors, nurses and laboratory health workers/ESY laboratories-university laboratories.

### 4.2 Sample

**Map 1:**
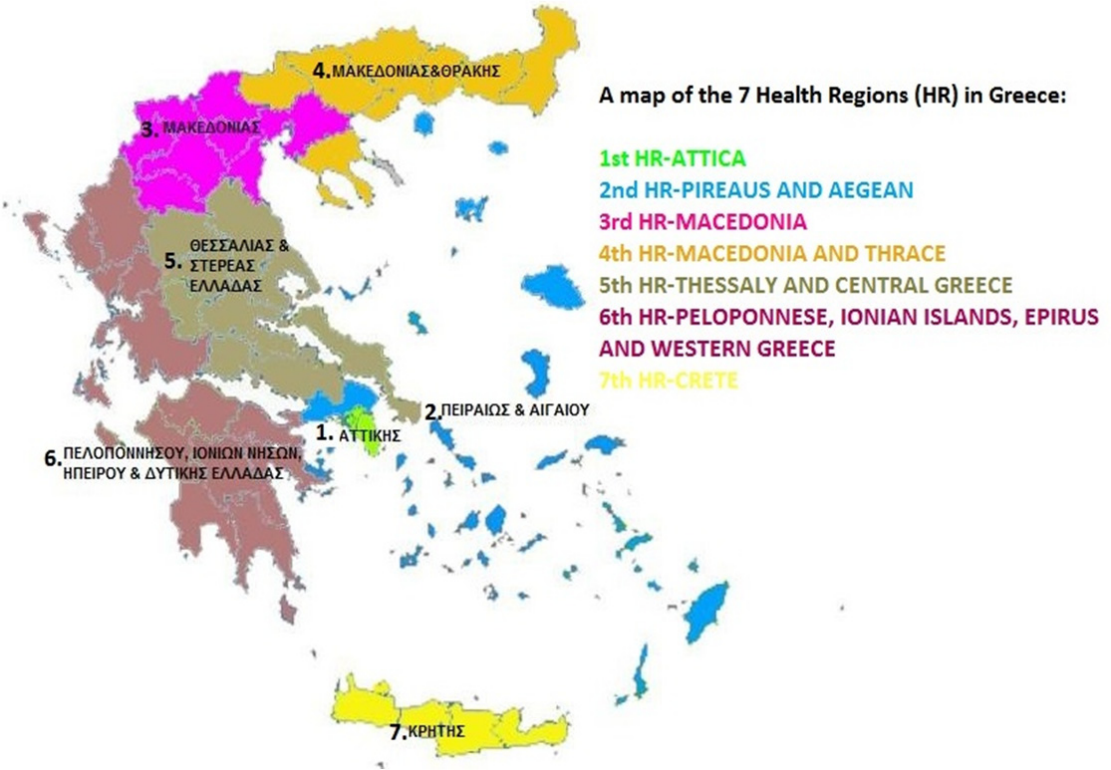
The 7 Health Regions of Greece

All secondary and tertiary public hospitals (ESY or University public hospitals) were included in the study (N=129). The reference population for calculating the supply and performance indicators was defined according to information collected and analyzed from the Hellenic Statistical Authority and each of the seven HRs. The total population of Greece in 2011 was 10.785.860, distributed in seven HRs (Health Regions) as follows:

1. The 1^st^ HR includes 4 geographic sub-regions with a population of 2.551.170.
2. The 2^nd^ HR includes 8 geographic sub-regions with a population of 1.767.580.
3. The 3^rd^ HR includes 2 geographic sub-regions with a population of 1.143.290.
4. The 4^th^ HR includes 5 geographic sub-regions with a population of 1.619.590.
5. The 5^th^ HR includes 2 geographic sub-regions with a population of 1.277.600.
6. The 6^th^ HR includes 4 geographic sub-regions with a population of 1.805.290.
7. The total population of the 7^th^ HR is 621.340. (Map 1)

## 5. Results

A. Supply Indicators for Hospital Clinics and Diagnostic Laboratories

A.1 Hospital beds/1000 population

The national average of hospital beds/1000 population is 2.9. The lowest indicator was recorded in the 5^th^ (2.04) and the 2^nd^ (2.32) HRs, whereas the highest in the 1^st^ (3.5), 7^th^ (3.45) and 3^rd^ (3.04) HRs (Table 1).

**Table 1:** General supply and performance indicators

Clinics (N)	Total	1^st^ HR		2^nd^ HR		3^rd^ HR		4^th^ HR		5^th^ HR		6^th^ HR		7^th^ HR	

	1581	417		229		168		228		153		271		115	
	
	Mean	Mean	Dev.	Mean	Dev.	Mean	Dev.	Mean	Dev.	Mean	Dev.	Mean	Dev.	Mean	Dev.
Hospital beds/1000 population	2.90	3.50	0.60	2.32	-0.58	3.04	0.14	3.03	0.13	2.04	-0.86	2.83	-0.07	3.45	0.55
Hospital Doctors/1000 population	1.42	1.99	0.57	1.09	-0.33	1.36	-0.06	1.41	-0.01	0.92	-0.50	1.26	-0.16	1.60	0.18
Hospital Nurses/1000 population	1.79	2.21	0.42	1.35	-0.44	1.75	-0.04	1.91	0.12	1.46	-0.33	1.71	-0.08	2.02	0.23
Total staff/1000 population	3.21	4.2	0.99	2.44	-0.77	3.11	-0.10	3.32	0.11	2.38	-0.83	2.97	-0.24	3.62	0.41
Hospital doctors/Hospital Bed	0.49	0.57	0.08	0.47	-0.02	0.45	-0.04	0.46	-0.03	0.45	-0.04	0.45	-0.04	0.46	-0.03
Hospital Nurses/Hospital Bed	0.62	0.63	0.01	0.58	-0.04	0.57	-0.05	0.63	0.01	0.71	0.09	0.60	-0.02	0.58	-0.04
Total staff/Hospital Bed	1.10	1.20	0.10	1.05	-0.05	1.02	-0.08	1.09	-0.01	1.16	0.06	1.05	-0.05	1.04	-0.06

*Note.* “Dev” refers to the deviance of the respective indicator from the overall average.

A.2 Hospital staff/1000 Population

As for doctors of hospital clinics/1000 population, the national average is 1.42. The highest indicator was recorded in the 1^st^ (1.99), 7^th^ (1.66) and 4^th^ (1.41) HRs, whereas the lowest in the 5^th^ and 2^nd^ HRs, 0.92 and 1.09 respectively. In terms of nurses, the national average was 1.79, with the best outcomes observed in the 1^st^ (2.21), 7^th^ (2.02) and 4^th^ (1.91) HRs, while the worst in the 5^th^ (1.46), 2^nd^ (1.35) and 3^rd^ HRs (1.75) (Table 1). As far as total staff/1000 population is concerned, the best results came from the 1^st^ (4.2), 7^th^ (3.62) and 4^th^ (3.32) HRs, while the worst came from the 5^th^ (2.38) and 2^nd^ (2.44) HRs.

A.3 Major Hospital Clinics per 100.000 Population

- The national average for internal medicine clinics (internal medicine) is 1.65/100.000 population. The best results were observed in the 1^st^ (2.04), 7^th^ (1.61), and 6^th^ (1.61), while the worst outcomes came from the 5^th^ and 3^rd^ HRs, 1.33 and 1.49 respectively.
- The national average for (general) surgery hospital clinics is 1.48/100.000 population. The highest concentrations were observed in the 1^st^ (1.76), 3^rd^ (1.57) and 4^th^ (1.54) HRs, while the lowest came from the 5^th^ (1.09), 2^nd^ (1.36) and 7^th^ (1.29) HRs.
- Cardiology hospital clinics present a 1.07 supply indicator as a national average, implying that the 6^th^ (1.33), 3^rd^ (1.22) and 7^th^ (1.13) HRs were amongst the best in this respect (i.e. clinics per 100.000 population. On the contrary, the 4^th^ (0.86), 2^nd^ (0.96) and 5^th^ (1.02) HRs were below the national average.
- The national average for pediatrics clinics is 0.77, with the best outcomes observed in the 7^th^ (1.13) and 6^th^ (1.05) HRs, and the worst in the 1^st^ (0.39) HR.
- Obstetrics-Gynecology has a 0.90 national average, with the best results in the 6^th^ (1.22), 7^th^ (1.13) and 3^rd^ (1.05) HRs. Again the worst result came from the 1^st^ HR (0.59).
- ICU have a 0.67 national average per 100.000 population, with the highest indicator recorded in the 7^th^ (0.96) HR and the lowest in the 5^th^ (0.39) HR.
- The national average for orthopedic clinics is 1.05 per 100.000 population. The highest (1.29) and lowest (0.73) indicators came from the 1^st^ and 2^nd^ HRs respectively.
- Pneumonology clinics present a national average of 0.48 clinics per 100.000 population. The best outcome was observed it the 7^th^ (0.8) and 1^st^ (0.78) HRs, whereas the worst in the 4^th^ and 5^th^ (0.31) HRs.
- Psychiatric clinics in Greek public hospitals present a national average of 0.40. The highest indicators come from the 2^nd^ (0.73), the 7^th^ (0.48) and the 4^th^ (0.43) HRs, and the lowest from the 5^th^ (0.16) and 1^st^ (0.31) HRs. The rather low scoring can be attributed to the fact that specialized psychiatric public hospitals are excluded from the research.
- Ophthalmology clinics present a national average of 0.74 clinics per 100.000 population with highest results in the 7^th^ (0.96), 3^rd^ (0.87), and 6^th^ (0.83) HRs. The lowest results appear in 2^nd^ (0.62) and 4^th^ (0.68) HRs.
- The otolaryngology (ENT) clinics of public Greek hospitals have a national average of 0.76 clinics per 100.000 population. The best outcomes came from the 3^rd^ (1.05), 7^th^ (0.96) and 6^th^ (0.83) HRs, while the worst from the 1^st^ (0.59) and 4^th^ (0.68) HRs.

A.4 Major Clinics’ Hospital Beds/1000 Population

- The national average for internal medicine (internal medicine) hospital beds is 0.51 per 1000 population. The best results were observed in the 1^st^ (1.76) HR, whereas the worst in the 5^th^ (0.38) HR.
- The national average for (general) surgery hospital beds is 0.3/1000 population. The highest concentration was observed in the 1^st^ (0.45), 3^rd^ (0.44) and 4^th^ (0.43) HRs, while the worst results came from the 5^th^ (0.38) and 3^rd^ (0.28) HRs.
- Cardiology hospital beds in Greece present a 0.24 per 1000 population supply indicator as a national average. The 1^st^ (0.28) and 7^th^ (0.28) HRs were the best in this respect whereas the 2^nd^ (0.19) and 5^th^ (0.18) were below the national average.
- The national average for pediatric hospital beds is 0.18 per 1000 population, with the best outcome observed in the 6^th^ (0.24), 7^th^ (0.23), and 4^th^ (0.22) HRs, and the worst in the 2^nd^ and 5^th^ (0.11) HRs.
- Obstetrics-Gynecology clinics have a 0.20 national average, with the best results in the 7^th^ (0.25), 6^th^ (0.25), and 4^th^ (0.23) HRs. Again the worst results came from the 2^nd^ (0.16) and 5^th^ (0.17) HRs.
- ICU hospital beds have a 0.67 national average, with the best results observed in the 1^st^ (0.08), 7^th^ (0.07), and 3^rd^ (0.07) HRs, while the worst came from the 5^th^ (0.03), 4^th^ (0.04) and the 2^nd^ (0.04) HRs.
- Hospital beds for orthopedic hospital clinics/1000 population have a national average of 0.24, with the highest observed in the 3^rd^ (0.32) HR, and the lowest in the 2^nd^ (0.15).
- Pneumonology hospital beds present a national average of 0.11 per 1000 population. Best and worst outcomes were observed in the 1^st^ (0.23) and 4^th^ (0.05) HRs respectively.
- Psychiatric beds in Greek public hospitals present a national average of 0.09. The highest scores belong to the 2^nd^ (0.27), and 7^th^ (0.11) HRs and the lowest to the 5^th^ (0.01) HR.
- Ophthalmology beds present a national average of 0.08 per 1000 population with the highest results in the 7^th^ (0.1) and 4^th^ (0.1) HRs. The lowest results appear in the 2^nd^ HR.
- The otolaryngology (ENT) beds score a national average of 0.08 per 1000 population. The best outcomes came from the 7^th^ (0.12) HR and the worst came from the 5^th^ (0.06).

A.5 Diagnostic Laboratory Staff/1000 population

As far as laboratory doctors/1000 population is concerned, the national average is 0.36 doctors/1000 population. The highest indicators belong to the 1st (0.55), 6^th^ (0.31) and 7^th^ (0.4) HRs, while the lowest to the 5^th^ (0.2) and 2^nd^ (0.27) HRs. Laboratory nurses have a national average of 0.12, with the highest results found in the 1^st^ (0.14) and 7^th^ (0.13) HRs, and the lowest in the 2^nd^ (0.02) HR. Laboratory health workers scored a 0.46 national average, with the highest results found in the 1^st^ (0.62), 7^th^ (0.53) and 4^th^ (0.47) HRs. The lowest results were located in the 5^th^ (0.35), and 2^nd^ (0.34) HRs. As far as the total lab staff is concerned, the best ratios for total staff/1000 population were observed in the 1^st^ (1.31) and 7^th^ (1.06) HRs, while the worst in the 2^nd^ (0.71) and 5^th^ (0.67) HRs.

A.6 Major Hospital Diagnostic Laboratories/100.000 population

The national average of laboratories/100.000 population is 6.14, with the highest in the 7^th^ (8.05), and 1^st^ (7.48) HRs. The lowest results were observed in the 5^th^ (4.7) and 2^nd^ (4.27) HRs. Results for each category of diagnostic laboratories per HR are presented in Table 2.

**Table 2:** Supply indicators: Major hospital diagnostic laboratories per 100.000 population for each HR and in total

Supply indicators (N)	Total	1^st^ HR		2^nd^ HR		3^rd^ HR		4^th^ HR		5^th^ HR		6^th^ HR		7^th^ HR	

	663	179		91		64		91		60		128		50	

	Mean	Mean	Dev.	Mean	Dev.	Mean	Dev.	Mean	Dev.	Mean	Dev.	Mean	Dev.	Mean	Dev.
Microbiology	1.16	1.02	-0.14	1.01	-0.15	1.22	0.06	1.11	-0.05	1.02	-0.14	1.6	0.44	1.29	0.13
Biochemistry	0.3	0.59	0.29	0.28	-0.02	0.08	-0.22	0.24	-0.06	0.08	-0.22	0.16	-0.14	0.64	0.34
Medical imaging	1.17	1.1	-0.07	1.02	-0.15	1.22	0.05	1.05	-0.12	1.01	-0.16	1.55	0.38	1.29	0.12
Hematology	0.66	0.82	0.16	0.51	-0.15	0.61	-0.05	0.62	-0.04	0.62	-0.04	0.66	0	0.8	0.14
Blood donor	0.79	0.74	-0.05	0.45	-0.34	1.05	0.26	0.74	-0.05	0.7	-0.09	1.63	0.84	0.8	0.01
Pathologoanatomy	0.64	0.82	0.18	0.4	-0.24	0.7	0.06	0.62	-0.02	0.39	-0.25	0.83	0.19	0.64	0
Cytology	0.44	0.7	0.26	0.34	-0.1	0.44	0	0.31	-0.13	0.15	-0.29	0.55	0.11	0.32	-0.12

*Note.* “Dev” refers to the deviance of the respective indicator from the overall average.

- Microbiology laboratories have a national average of 1.16/100.000 population, with the highest indicators in the 6^th^ (1.6), 7^th^ (1.29) and 3^rd^ (1.22) HRs, and the lowest in the 1^st^ (1.02) and 2^nd^ (1.01) HRs.
- Biochemistry laboratories have a national average of 0.3, with the highest observed in the 7^th^ (0.64) and 1^st^ (0.59) HRs and the lowest in the 3^rd^ (0.08) and 5^th^ (0.08) HRs.
- Medical Imaging laboratories have a national average of 1.17, with the best outcome observed in the 6^th^ (1.55) and 7^th^ (1.55) HR and the lowest in the 1^st^ (1.1).
- Hematology laboratories have a national average of 0.66, with the highest results in the 1^st^ (0.82) and 7^th^ (0.8) HRs, while the lowest at the 2^nd^ (0.51) and the 3^rd^ (0.61) HRs.
- Blood donor labs have a national average of 0.79 with the highest results found in the 6^th^ (1.63) and 3^rd^ (1.05) HRs, and the lowest in the 2^nd^ (0.45) HR.
- Pathologoanatomy laboratories have a national average of 0.64 labs/100.000 population with the highest ratios found in the 6^th^ (0.83) and 1^st^ (0.82) HRs, and the worst in the 5^th^ (0.39) and 2^nd^ (0.40) HRs.
- Finally, cytology laboratories present a national average of 0.44 laboratories per 100.000 population, with the highest results found in the 1^st^ (0.70) and 6^th^ (0.55) HRs, and the lowest in the 5^th^ (0.15) HR.

B. Performance Indicators for Hospital Clinics and Diagnostic Laboratories

B.1 Hospital Staff/Hospital Bed

The national average of hospital doctors per hospital bed is 0.49. The worst outcomes were observed in the 3^rd^, 5^th^, and 6^th^ HRs with 0.45 and in the 4^th^ and 7^th^ regions with 0.46 doctors per hospital bed. As a result only the 1^st^ HR has a rather good outcome with 0.57 doctors per hospital bed. In terms of nurses, the national average is 0.62 nurses/hospital bed, with the best outcomes observed in the 5^th^ (0.71) HR, while the worst outcomes come from the 3^rd^ (0.57) and 7^th^ (0.58). As far as total staff per hospital bed is concerned, best results came from the 1^st^ (1.20), and 5^th^ (1.16) HRs, while the worst from the 3^rd^ (1.02) and 7^th^ (1.04) (Table 1).

B.2 Major Clinics Staff/Hospital Bed (Tables 3-4)

**Table 3:** Performance indicators-major clinics’ doctors per hospital bed for each health region and in total

Performance Indicators	Total	1^st^ HR		2^nd^ HR		3^rd^ HR		4^th^ HR		5^th^ HR		6^th^ HR		7^th^ HR	

	Mean	Mean	Dev.	Mean	Dev.	Mean	Dev.	Mean	Dev.	Mean	Dev.	Mean	Dev.	Mean	Dev.
Internists/Bed	0.52	0.51	-0.01	0.5	-0.02	0.5	-0.02	0.56	0.04	0.58	0.06	0.51	-0.01	0.48	-0.04
Surgery doctors/Bed	0.46	0.47	0.01	0.54	0.08	0.44	-0.02	0.45	-0.01	0.45	-0.01	0.44	-0.02	0.44	-0.02
Cardiology doctors/Bed	0.49	0.62	0.13	0.52	0.03	0.37	-0.12	0.4	-0.09	0.43	-0.06	0.47	-0.02	0.43	-0.06
Pediatrics doctors/Bed	0.56	0.67	0.11	0.61	0.05	0.47	-0.09	0.53	-0.03	0.55	-0.01	0.48	-0.08	0.65	0.09
ObGyn doctors/Bed	0.41	0.48	0.07	0.42	0.01	0.44	0.03	0.39	-0.02	0.35	-0.06	0.37	-0.04	0.39	-0.02
ICU doctors/Bed	1.08	1.12	0.04	1.22	0.14	0.85	-0.23	1.15	0.07	1.13	0.05	0.97	-0.11	1.15	0.07
Orthopedics doctors/Bed	0.43	0.58	0.15	0.46	0.03	0.47	0.04	0.33	-0.1	0.38	-0.05	0.38	-0.05	0.35	-0.08
Pneumonology doctors/Bed	0.45	0.45	0	0.37	-0.08	0.44	-0.01	0.54	0.09	0.49	0.04	0.42	-0.03	0.47	0.02
Psychiatrics doctors/Bed	0.39	1.06	0.67	0.14	-0.25	0.55	0.16	0.48	0.09	0.75	0.36	0.54	0.15	0.4	0.01
Ophthalmology doctors/Bed	0.7	0.86	0.16	1.04	0.34	0.63	-0.07	0.6	-0.1	0.49	-0.21	0.64	-0.06	0.54	-0.16
Otolaryngology doctors/Bed	0.56	0.67	0.11	0.6	0.04	0.55	-0.01	0.43	-0.13	0.58	0.02	0.51	-0.05	0.51	-0.05

*Note.* “Dev” refers to the deviance of the respective indicator from the overall average.

**Table 4:** Performance indicators: Major clinics’ nurses per hospital bed for each health region and in total

Performance Indicators	Total	1^st^ HR		2^nd^ HR		3^rd^ HR		4^th^ HR		5^th^ HR		6^th^ HR		7^th^ HR	

	Mean	Mean	Dev.	Mean	Dev.	Mean	Dev.	Mean	Dev.	Mean	Dev.	Mean	Dev.	Mean	Dev.
Internal Medicine nurses/Bed	0.52	0.51	-0.01	0.5	-0.02	0.5	-0.02	0.56	0.04	0.58	0.06	0.51	-0.01	0.48	-0.04
Surgery nurses/Bed	0.53	0.48	-0.05	0.64	0.11	0.49	-0.04	0.53	0	0.61	0.08	0.54	0.01	0.48	-0.05
Cardiology nurses/Bed	0.73	0.81	0.08	0.69	-0.04	0.67	-0.06	0.75	0.02	0.77	0.04	0.7	-0.03	0.56	-0.17
Pediatrics nurses/Bed	0.46	0.54	0.08	0.45	-0.01	0.5	0.04	0.41	-0.05	0.59	0.13	0.43	-0.03	0.44	-0.02
Ob-Gyn nurses/Bed	0.69	0.63	-0.06	0.6	-0.09	0.72	0.03	0.8	0.11	0.76	0.07	0.67	-0.02	0.69	0
ICU nurses/Bed	2.92	2.76	-0.16	2.88	-0.04	2.15	-0.77	3.8	0.88	3.62	0.7	2.93	0.01	2.98	0.06
Orthopedics nurses/Bed	0.42	0.52	0.1	0.3	-0.12	0.5	0.08	0.44	0.02	0.37	-0.05	0.42	0	0.3	-0.12
Pneumonology nurses/Bed	0.47	0.48	0.01	0.33	-0.14	0.49	0.02	0.47	0	0.79	0.32	0.48	0.01	0.44	-0.03
Psychiatrics nurses/Bed	0.76	1.22	0.46	0.65	-0.11	0.61	-0.15	0.69	-0.07	1.45	0.69	0.74	-0.02	0.83	0.07
Opthalmology nurses/Bed	0.41	0.53	0.12	0.48	0.07	0.31	-0.1	0.33	-0.08	0.37	-0.04	0.4	-0.01	0.28	-0.13
Otolaryncology nurses/Bed	0.38	0.37	-0.01	0.32	-0.06	0.29	-0.09	0.45	0.07	0.34	-0.04	0.42	0.04	0.47	0.09

*Note.* “Dev” refers to the deviance of the respective indicator from the overall average.

- Internal medicine clinics
  As for internists/hospital bed, the national average is 0.5 with the highest outcomes observed in the 1^st^ (0.54), 4^th^ (0.52) and 7^th^ (0.51) HRs. The lowest outcomes were observed in the 2^nd^ (0.48), 3^rd^ (0.47), 5^th^ (0.47) 6^th^ (0.45) HRs. Internal medicine nurses have a national average of 0.52, with best results in the 5^th^ (0.58) and 4^th^ (0.56) HRs, and worst in the 2^nd^ (0.5), 3^rd^ (0.5) and 7^th^ (0.48) HRs. As for total staff, internal medicine clinics present best results in the 1^st^ (1.05), 4^th^ (1.08) and 5^th^ (1.05) HRs, and worst in the 2^nd^ (0.98), 3^rd^ (0.97) and 6^th^ (0.96) HRs.
- (General) Surgery Clinics
  The national average of surgeons is 0.46, with better results coming from the 2^nd^ (0.54) HR, and worst from the 3^rd^ (0.44), 6^th^ (0.44), and 7^th^ (0.44) HRs. Surgery nurses are at a national average of 0.53, with the best results in the 2^nd^ (0.64) and 5^th^ (0.61) HRs, and the worst in the 1^st^ (0.48), 3^rd^ (0.49) and 7^th^ (0.48) HRs. As far as total staffing of surgery clinics is concerned, best results can be found in the 2^nd^ (1.18) and 5^th^ (1.06) HRs, and worst in the 1^st^ (0.95), 3^rd^ (0.93) and 7^th^ (0.92) HRs.
- Cardiology Clinics
  The national average of cardiologists per bed is 0.49. The best results came from the 1^st^ (0.62) and the 2^nd^ (0.52) HRs, and the worst from the 3^rd^ (0.37) HR. Cardiology clinic nurses are at a national average of 0.73. Best results were observed in the 1^st^ (0.81) and 5^th^ (0.77) HRs, and worst in the 3^rd^ (0.67) and 7^th^ (0.56) HRs. As far as the total staff of cardiology clinics/bed is concerned, the best indicator results comes from the 1^st^ (1.43) and 2^nd^ (1.21) HRs and the worst from the 3^rd^ (1.04) and 7^th^ (0.99) HRs.
- Pediatrics
  The national average of pediatricians is 0.56. The highest ratio is in the 1^st^ (0.67), 2^nd^ (0.61) and 7^th^ (0.65) HRs, and the lowest in the 3^rd^ (0.47) and 6^th^ (0.48). Pediatric nurses per hospital bed are on average 0.46. The best results in this respect come from the 5^th^ (0.59), 1^st^ (0.54) and 3^rd^ (0.5) HRs, while the worst from the 4^th^ (0.41) and 6^th^ (0.43) HRs. As for total staff/bed, the best indicator is evident in the 1^st^ (1.21) and 5^th^ (1.14), while the worst in the 3^rd^ (0.97), 4^th^ (0.94), and 6^th^ (0.91) HRs.
- Obstetrics – Gynecology clinics
  Obstetrics/gynecology doctors per bed are at a national average of 0.41. The best ratios are observed in the1^st^ (0.48) and 3^rd^ (0.44) HRs, and the worst in the 5^th^ (0.35) and 6^th^ (0.37). The national average of nurses in obstetrics-gynecology is 0.69. Thus, HRs exceeding the national average are the 4^th^ (0.8) and 5^th^ (0.7), whereas those lagging are the 2^nd^ (0.62) and 6^th^ (0.6). As for overall staff, the best indicators are from the 4^th^ (1.19) and 3^rd^ (1.16) HRs, while the worst from the 2^nd^ (1.02) and 6^th^ (1.04) HRs.
- Intensive Care Units
  Intensive care doctors per hospital bed are 1.08. The best outcomes were observed in the 2^nd^ (1.22) HR, and the lowest in the 3^rd^ (0.85). The national average for nurses is 2.92, with the highest results found in the 4^th^ (3.8) and 5^th^ (3.62) HRs. The worst HR was the 3^rd^ (2.15). As for total staff, the best results came from the 4^th^ (4.95), 5^th^ (4.75), and 7^th^ (4.13) HRs, while the worst from the 3^th^ (3.0) and 6^th^ (3.9) HRs.
- Orthopedics
  The national average of orthopedics doctors per hospital bed is 0.43. The best results were found in the 1^st^ (0.58) HR, and the worst in the 4^th^ (0.33). Orthopedics nurses are 0.42 per hospital bed. The best outcomes can be found in the 1^st^ (0.52), 3^rd^ (0.5) and 4^th^ (0.44) HRs, and the worst in the 2^nd^ (0.3) and 7^th^ (0.3). When adding the number of doctors and nurses, the best indicators is in the 1^st^ (1.1), 3^rd^ (0.97) and 6^th^ (0.8) HRs, and the worst in the 5^th^ (0.2), 2^nd^ (0.76), and 7^th^ (0.65) HRs.
- Pneumonology clinics
  The average number of pneumonologists per hospital bed is 0.45. The highest ratio is in the 4^th^ (0.54) HR and the lowest in the 2^nd^ (0.37). Nursing staff have a national average of 0.47, which is exceeded by the 5^th^ (0.79), 3^rd^ (0.49) and 1^st^ (0.48) HRs. As far as total staff is concerned, the best results were observed in the 4^th^ (1.01) and 5^th^ (1.28) HRs, and the worst in the 2^nd^ (0.70) and 6^th^ (0.9) HRs.
- Psychiatric clinics
  The national average of psychiatrists per hospital bed is 0.39. HRs exceeding this average were the 1^st^ (1.06), 5^th^ (0.75) and 3^rd^ (0.55). The worst HR in terms of deviation from the national average was the 2^nd^ (0.14). The national average of nurses/psychiatric beds is 0.76, with best outcomes found in the 1^st^ (1.22) and 5^th^ (1.45) HRs, and the worst in the 2^nd^ and 3^rd^, with 0.65 and 0.61 respectively. As for doctors and nurses combined, the best outcomes come from the 1^st^ (2.28) HR, and the worst from the 2^nd^ (0.79).
- Ophthalmology clinics
  Ophthalmologists are at a national average ratio of 0.7 and HRs exceeding this figure are the 1st (0.85) and 2^nd^ (1.04). Those lagging are the 5^th^ (0.49) and 7^th^ (0.54) HRs. As for nurses, their national average is 0.41. Again the best results for this indicator were found in the 1^st^ (0.53), and 2^nd^ (0.48) HRs, while the worst in the 7^th^ (0.28), 3^rd^ (0.31) and 4^th^ (0.33). As for doctors and nurses combined, best outcomes were observed in the 1^st^ (1.39) and 2^nd^ (1.52) HRs, while the worst in the 5^th^ (0.86) and 7^th^ (0.82) HRs.
- Otolaryngology clinics
  Otolaryngologists (ENTs) have a national average of 0.56. HRs exceeding this limit are the 1^st^ (0.67) and 2^nd^ (0.6), and regions falling back are the 4^th^ (0.43), 6^th^ (0.51) and 7^th^ (0.51). As for nurses, their national average is 0.38. Again the best results for this indicator were found in the 7^th^ (0.47), 4^th^ (0.45) and 6^th^ (0.42) HRs, while the worst in the 2^nd^ (0.32), 3^rd^ (0.29) and 5^th^ (0.34). As for overall staff, best results were observed for the 1^st^ (1.04) and 7^th^ (0.98) HRs, and worst for the 3^rd^ (0.84), 4^th^ (0.88), and 2^nd^ (0.92) HRs.

## 6. Discussion

Starting from hospital beds per 1.000 population, even though OECD and EU-15 countries seem to do better than Greece in this ratio (4.8 and 5.3 respectively), one has to take into account that their respective indicators have been calculated with the inclusion of private hospital beds (OECD, 2013; WHO Regional Office for Europe, 2011). If private hospital beds were included in the present study, the hospital beds/1000 population indicator for Greece would increase to approximately 5 hospital beds (70% public and 30% private) per 1000 population. As a result, the indicator is further adjusted to 3.5 public hospital beds per 1000 population, including psychiatric hospital beds. Thus, HRs that surpass 3 hospital beds per 1000 population, perform rather well when compared to international standards, while the rest are required to add more hospital beds. Alternatively, a transfer of hospital beds is suggested according to two criteria, geographical proximity and the surplus of hospital beds in some HRs. According to the analysis, most HRs are above the national benchmark of 3 hospital beds/1000 population, and those lagging are the 2^nd^, 5^th^ and 6^th^ (though the latter is borderline, i.e. 2.83). Thus it is proposed, based on this research, to reduce beds in the 1^st^ (-12%), 3^rd^ (-8%) and 7^th^ (-9%) HRs and to increase through transfers those in the 2^nd^ (especially in the Aegean Islands) and 5^th^ HRs (Table 5).

**Table 5:** Reengineering or reshuffling of staff or staff transfers according to hospital beds for each health region and in total

(HB)/Staff	N	1^st^ HR			2^nd^ HR			3^rd^ HR			4^th^ HR			5^th^ HR			6^th^ HR			7^th^ HR		

		(S)	(E)	(A)	(S)	(E)	(A)	(S)	(E)	(A)	(S)	(E)	(A)	(S)	(E)	(A)	(S)	(E)	(A)	(S)	(E)	(A)
(HB)	31297	7426	8952	-1526	5149	4098	1051	3339	3473	-134	4720	4909	-189	3583	2613	970	5258	5108	150	1822	2144	-322
(HD)	15313	3622	5077	-1455	2509	1934	575	1622	1561	61	2300	2292	8	1814	1177	637	2564	2278	286	882	994	-112
(HN)	19320	4569	5630	-1061	3166	2394	772	2048	2000	48	2901	3089	-188	2289	1863	426	3233	3091	142	1114	1253	-139
Total (HS)	34633	15617	19659	-4042	10824	8426	2398	7009	7034	-25	9921	10290	-369	7686	5653	2033	11055	10477	578	3818	4391	-573

(S):Suggested, (E):Existing, (A):Added; (HB): Hospital Beds, (HD):Hospital Doctors, (HN):Hospital Nurses, Total (HS):Total Hospital Staff.

Supplementary to the above, it is important to note that it is proposed that relative changes be planned within the hospitals concerning the clinics-departments. Indicatively, in the 1^st^ HR pneumonology clinics should be merged from 20 to 12 (and the beds respectively), while gynecology and pediatric clinics should be developed at selected large hospitals (redistributing existing beds). In the 2^nd^ HR, psychiatric clinics should be merged from 13 to 7 (and the beds respectively), while orthopedic, pneumonology and ophthalmology clinics should be developed at selected hospitals (and the beds respectively on the islands). In the 3^rd^ HR, ENT clinics should be merged from 12 to 9, while orthopedic beds should be reduced and psychiatric beds should be increased. In the 4^th^ HR pneumonology clinics should be increased from 5 to 10 (and the beds respectively), and cardiology – orthopedic clinics from 14 to 17 (redistributing existing beds). In the 5^th^ HR psychiatric clinics should be increased from 2 to 5 and ICU from 5 to 8 (and the beds respectively). In the 6^th^ HR gynecology and pediatric clinics should be merged from 22 to 16 and from 19 to 14 respectively, while pneumonology clinics should be increased from 6 to 9 (and the beds respectively). Finally, in the 7^th^ HR the proposal concerns a reduction of pneumonology and pediatric beds.

Concerning human resources ratios, the HRs exceeding 1.3 NHS doctors per 1.000 population score rather well, while the rest need to hire more doctors or transfer doctors from a nearby HR, that exceed 1.3 doctors per 1000 population, such as the 1^st^, the 3^rd^, the 4^th^ and the 6^th^ HRs. On the same line of analysis, HRs that need urgent staff support are the 2^nd^, and mostly the 5^th^ HRs (Table 5). The issue of 2^nd^ RHA is complicated because of many islands and needs further consideration concerning HR transfers. 5^th^ RHA must be supported mainly from 3^rd^ and 4^th^. The HRs that exceed the ratio of 1.7 nursing staff per 1.000 population are performing rather well, while regions below this threshold need to be supported with additional nursing staff. Alternatively to hiring around 3000 nursing staff, it is suggested to reshuffle nurses between the regions that are close enough and exceed or lag from the national average. According to the analysis of the raw data collected from the public hospitals all over the country, the 1^st^, 4^th^, 7^th^, and marginally the 3^rd^ (1.75) and the 6^th^ (1.71) fulfill the threshold of 1.7 nurses per 1000 population. The ones that fail to reach the threshold, the 2^nd^ and the 5^th^, need support from either the 1^st^ or the 3^rd^ and the 4^th^ HR respectively. The situation in regard to other (non-medical and non-nursing hospital staff) is almost similar (Table 5).

Specific reference should be made for the reengineering of ESY hospital laboratories. Mergers are needed in the 1^st^, 6^th^ and 7^th^ HR, especially in biochemistry (1^st^ and 7^th^) and microbiology, as well as in medical imaging labs (6^th^). The remaining HR need selected improvements, e.g. the 2^nd^ HR (blood banks and pathologoanatomy labs), the 4^th^ HR (cytology labs) and a few biochemistry labs in the 3^rd^ and 5^th^ HR (Table 6).

**Table 6:** Reengineering of reshuffling of hospital laboratories per health region

Laboratories/HR	Total (N=561)	1^st^ HR			2^nd^ HR			3^rd^ HR			4^th^ HR			5^th^ HR			6^th^ HR			7^th^ HR		

		(S)	(E)	(A)	(S)	(E)	(A)	(S)	(E)	(A)	(S)	(E)	(A)	(S)	(E)	(A)	(S)	(E)	(A)	(S)	(E)	(A)
Microbiology	126	30	26	4	20	18	2	14	14	0	19	18	1	15	13	2	21	29	-8	7	8	-1
Biochemistry	33	9	15	-6	5	5	0	3	1	2	5	4	1	4	1	3	5	3	2	2	4	-2
Medical imaging	126	30	28	2	21	18	3	13	14	-1	19	17	2	15	13	2	21	28	-7	7	8	-1
Hematology	72	18	21	-3	12	9	3	7	7	0	11	10	1	8	8	0	12	12	0	4	5	-1
Blood donor	86	21	19	2	14	8	6	9	12	-3	13	12	1	10	9	1	14	21	-7	5	5	0
Pathologoanatomy	70	17	21	-4	12	7	5	8	8	0	10	10	0	8	5	3	11	15	-4	4	4	0
Cytology	48	11	18	-7	8	6	2	5	5	0	7	5	2	6	2	4	8	10	-2	3	2	1

Finally tables 7 and 8 present the reengineering and reshuffling of hospital doctors and nurses of the hospital clinics-departments of the main specialties. Transfers of doctors are proposed from the 1^st^ HR (Athens) to the 2^nd^ HR (mainly islands), orthopedic doctors from the 3^rd^ to the 4^th^ HR and internal medicine doctors from the 4^th^ to the 3^rd^ HR (both mainly within Salonika), general surgery doctors from the 3^rd^ and 4^th^ to the 5^th^ HR, which needs doctors in other specialties as well (transferred from other HRs). On the other hand, nurses’ shortage is obvious all over the country. Because of the current restrictions to hire new personnel, as a result of the economic crisis, our suggestion is a selected transfer of nurses between neighboring HRs like 1^st^ to 2^nd^, 4^th^ to 3^rd^ and 6^th^ to 5^th^.

**Table 7:** Reengineering or reshuffling of hospital doctors of major hospital clinics per health region and in total

Hospital doctors of major clinics (N)	Total (N) Doctors	1^st^ HR			2^nd^ HR			3^rd^ HR			4^th^ HR			5^th^ HR			6^th^ HR			7^th^ HR		

		(S)	(E)	(A)	(S)	(E)	(A)	(S)	(E)	(A)	(S)	(E)	(A)	(S)	(E)	(A)	(S)	(E)	(A)	(S)	(E)	(A)
Internal Medicine	2726	647	814	-167	446	396	50	288	258	30	409	428	-19	322	233	89	457	452	5	157	145	12
Surgery	1927	454	539	-85	316	272	44	205	222	-17	288	323	-35	229	160	69	321	315	6	114	96	18
Cardiology	1275	302	450	-148	209	173	36	135	121	14	192	158	34	151	100	51	213	199	14	73	74	-1
Pediatrics	1085	256	278	-22	178	122	56	116	108	8	164	191	-27	129	82	47	181	211	-30	61	93	-32
Ob-Gyn	895	211	224	-13	147	123	24	96	101	-5	134	143	-9	106	77	29	149	166	-17	52	61	-9
ICU	660	150	230	-80	107	99	8	73	68	5	99	83	16	81	51	30	108	77	31	42	52	-10
Orthopedics	1096	260	380	-120	179	122	57	114	136	-22	164	127	37	129	106	23	186	161	25	64	64	0
Pneumonology	545	126	267	-141	88	38	50	58	59	-1	83	45	38	69	31	38	90	61	29	31	44	-13
Psychiatrics	387	92	126	-34	63	70	-7	41	40	1	58	60	-2	46	15	31	64	47	17	23	29	-6
Ophthalmology	620	147	210	-63	102	77	25	66	68	-2	93	97	-4	73	51	22	103	83	20	36	34	2
Otolaryngology	526	121	165	-44	86	81	5	57	62	-5	79	68	11	64	48	16	87	64	23	32	38	-6

(S): Suggested, (E): Existing, (A):Added.

**Table 8:** Reengineering or reshuffling of hospital nurses of major hospital clinics per health region and in total

Hospital nurses of major clinics (N)	Total (N) Nurses	1^st^ HR			2^nd^ HR			3^rd^ HR			4^th^ HR			5^th^ HR			6^th^ HR			7^th^ HR		

		(S)	(E)	(A)	(S)	(E)	(A)	(S)	(E)	(A)	(S)	(E)	(A)	(S)	(E)	(A)	(S)	(E)	(A)	(S)	(E)	(A)
Internal Medicine	2854	676	760	-84	467	419	48	303	279	24	429	456	-27	338	287	51	478	515	-37	163	138	25
Surgery	2218	521	558	-37	363	320	43	237	245	-8	333	370	-37	264	217	47	371	402	-31	129	106	23
Cardiology	1903	449	586	-137	312	229	83	202	221	-19	287	294	-7	226	182	44	318	294	24	109	97	12
Pediatrics	896	211	224	-13	147	90	57	95	96	-1	135	148	-13	106	88	18	150	187	-37	52	63	-11
Ob-Gyn	1504	354	294	60	246	173	73	160	172	-12	226	294	-68	179	163	16	251	301	-50	88	107	-19
ICU	1774	404	565	-161	286	234	52	196	172	24	266	274	-8	216	163	53	292	232	60	114	134	-20
Orthopedics	1084	257	343	-86	178	78	100	114	149	-35	163	171	-8	128	105	23	182	182	0	62	56	6
Pneumonology	576	134	281	-147	94	34	60	63	62	1	87	38	49	72	50	22	94	70	24	32	41	-9
Psychiatrics	749	177	144	33	123	322	-199	81	44	37	113	85	28	89	29	60	123	65	58	43	60	-17
Ophthalmology	362	86	130	-44	59	36	23	39	33	6	54	54	0	43	38	5	60	53	7	21	18	3
Otolaryngology	353	83	90	-7	57	43	14	38	33	5	53	71	-18	42	28	14	58	53	5	22	35	-13

(S): Suggested, (E): Existing, (A): Added.

This research has its own limitations. First of all it may need further examination of the statistics and secondly further evaluation of local needs. However, policy makers have a tool that combines elements of supply (ESY) and demand (population). This effort should be combined with past proposals in order to improve distribution of units, beds and staff, mainly within HRs and inside the hospitals. This rationality of resources will decrease operational costs and secure better access to the population. We also hope that our research enhances the scientific dialogue among the various experts and institutions of the country and abroad to the reengineering issue of merging through redistribution.
